# Kinase inhibitors increase individual radiation sensitivity in normal cells of cancer patients

**DOI:** 10.1007/s00066-022-01945-y

**Published:** 2022-04-26

**Authors:** Tina Jost, Barbara Schuster, Lucie Heinzerling, Thomas Weissmann, Rainer Fietkau, Luitpold V. Distel, Markus Hecht

**Affiliations:** 1 Department of Radiation Oncology, University Hospital Erlangen, Friedrich-Alexander-Universität Erlangen-Nürnberg, Erlangen, Germany; 2 Comprehensive Cancer Center Erlangen-EMN, Erlangen, Germany; 3 Clinic and Polyclinic for Dermatology and Allergology, University Hospital München, Ludwig-Maximilian-Universität (LMU), Munich, Germany

**Keywords:** Fluorescence in situ hybridization, Blood, Small molecules, Targeted therapy, Chromosomal aberrations

## Abstract

**Purpose:**

Kinase inhibitors (KI) are known to increase radiosensitivity, which can lead to increased risk of side effects. Data about interactions of commonly used KI with ionizing radiation on healthy tissue are rare.

**Patients and methods:**

Freshly drawn blood samples were analyzed using three-color FISH (fluorescence in situ hybridization) to measure individual radiosensitivity via chromosomal aberrations after irradiation (2 Gy). Thresholds of 0.5 and 0.6 breaks/metaphase (B/M) indicate moderate or clearly increased radiosensitivity.

**Results:**

The cohorts consisted of healthy individuals (NEG, *n* = 219), radiosensitive patients (POS, *n* = 24), cancer patients (*n* = 452) and cancer patients during KI therapy (*n* = 49). In healthy individuals radiosensitivity (≥ 0.6 B/M) was clearly increased in 5% of all cases, while in the radiosensitive cohort 79% were elevated. KI therapy increased the rate of sensitive patients (≥ 0.6 B/M) to 35% significantly compared to 19% in cancer patients without KI (*p* = 0.014). Increased radiosensitivity of peripheral blood mononuclear cells (PBMCs) among patients occurred in six of seven KI subgroups. The mean B/M values significantly increased during KI therapy (0.47 ± 0.20 B/M without compared to 0.50 ± 0.19 B/M with KI, *p* = 0.047).

**Conclusions:**

Kinase inhibitors can intensify individual radiosensitivity of PBMCs distinctly in 85% of tested drugs.

## Introduction

Targeted drug therapy with various kinase inhibitors is becoming increasingly important in the treatment of cancer patients. Tyrosine kinase inhibitors such as alectinib, crizotinib and osimertinib are relevant in the treatment of non-small cell lung cancer (NSCLC), which represents 85% of all lung cancer cases [1]. Oncogenic drivers like epidermal growth factor receptor (EGFR) are found in 10–15% and for anaplastic lymphoma kinase (ALK) in up to 3–7% of patients [2]. In a recent phase III trial median overall survival (OS) of the chemotherapy approach was 47.5 months, whereas this endpoint in the KI group was not reached after a median follow-up duration of 70 months [3]. Further studies showed advantage of second-generation inhibitor alectinib vs. crizotinib with a rate of investigator-assessed progression-free survival (PFS) of 68.4% vs. 48.7% after 12 months, respectively [4]. Furthermore, in cases of renal cancer, tyrosine kinase inhibitor pazopanib, a multikinase inhibitor targeting vascular endothelial growth factor receptors (VEGFs), is very efficient in first-line treatment [5].

Previous studies found increased individual radiosensitivity in cancer patients compared to healthy individuals [6]. Beside this, different drugs such as the BRAF kinase inhibitor vemurafenib have radiosensitizing potential resulting in higher rates of severe side effects like radiodermatitis ≥ 2° during radiotherapy (RT) with BRAF inhibitor therapy (44%) vs. 8% without KI (*p* = 0.004) [7, 8]. Consequently, withholding kinase inhibitor therapy ≥ 3 days before and after fractionated RT and withholding ≥ 1 day pre- and poststereotactic radio surgery (SRS) is recommended by the Eastern Cooperative Oncology Group (ECOG) [9]. However, recent data indicate an improvement of local tumor control when kinase inhibitor therapy is combined with intracranial stereotactic RT without an increase in necrosis rates [10].

Regarding BRAF inhibitors (BRAFi) like vemurafenib and dabrafenib, cellular mechanisms of radiosensitization are partly known. In thyroid cancer BRAFV600E mutations promote nonhomologous end joining activity, a major pathway of DNA double strand break repair [11]. Currently, few data are available for most kinase inhibitors and their possibly radiosensitizing potential [12]. Groups like Falcão et al. [13] used peripheral blood lymphocytes and cancer cell lines for comparison of RT effects and Cheng et al. [14] reported a correlation between prostate cancer cell line and blood of cancer patients. Finally, Keller et al. (2015) demonstrated that increased radiosensitivity correlates with HIV‑1 treatment containing non-nucleoside reverse transcriptase inhibitors (NNRTI) in PBMCs of a specific subgroup of patients in addition to experimental in vitro data of cancer cell lines and healthy fibroblasts showing reduced survival fraction and increased activation of DNA repair proteins like H2AX, ATM, Nbs and 53BP1. Regarding the resilient correlation of radiosensitivity of blood lymphocytes and in vitro data of cancer cell lines [15], we investigated blood samples of cancer patients treated with different tyrosine and serine/threonine kinase inhibitors. Citing Hasan Murshed in *Fundamentals of Radiation Oncology* [16] and Furgason and el Bahassi [17], increased radiosensitivity is considered to be an advantage for improved local tumor control. At the same time the risk of a possible severe therapy-related sequelae in the healthy surrounding tissue should also be taken into account. To cover a wide range of outcomes a heterogeneous patient cohort of eight different entities, including rectal, lung, breast, prostate and others, was used. The influence on individual radiosensitivity of normal tissue by simultaneous kinase inhibitor treatment was studied using a three-color FISH analysis approach. The radiosensitivity was determined based on the rates of chromosomal aberrations, calculated as B/M after ex vivo irradiation of peripheral blood lymphocytes as an indicator of effects on healthy tissue.

## Materials and methods

### Patients and study design

Peripheral blood lymphocytes of healthy individuals and cancer patients were analyzed by a three-color FISH method to measure individual radiosensitivity. A historical cohort consisting of healthy individuals served as negative control. Selection criteria was “no prior or concurrent malignancies” and a Karnofsky performance status score of at least 90. The cohort was stratified by age. A historical cohort of cancer patients served as the control cohort. Patients with remarkable radiation-related chronic toxicity of ≥ grade 3 according to the Radiation Therapy Oncology Group (RTOG), e.g., fibrosis (grade 3 or 4) and bladder contracture after irradiation, were defined as “sensitive” to ionizing radiation (IR) and used as positive control [6, 18, 19]. In the current study, blood samples of patients with systemic therapy with kinase inhibitors were collected to study their individual radiosensitivity.

For this open cohort study, patients were collected consecutively between 2018 and 2020 at the radiation oncology department of the University Hospital Erlangen. Collection was prospective and a total of 49 patients having kinase inhibitor treatment were included. Inclusion criteria were the following: having cancer, being treated with a kinase inhibitor, age over 18 and written informed consent of the participate. Blood samples were taken during continuous inhibitor treatment.

### Trial oversight

The institutional review board at Friedrich-Alexander-Universität Erlangen-Nürnberg approved the study (No. 21_19 B). The study was performed in accordance with the Declaration of Helsinki. All patients gave written informed consent that comprised a data privacy clause for data collection and analysis for research purposes.

### Three-color fluorescence in situ hybridization

**Fig. 1:**
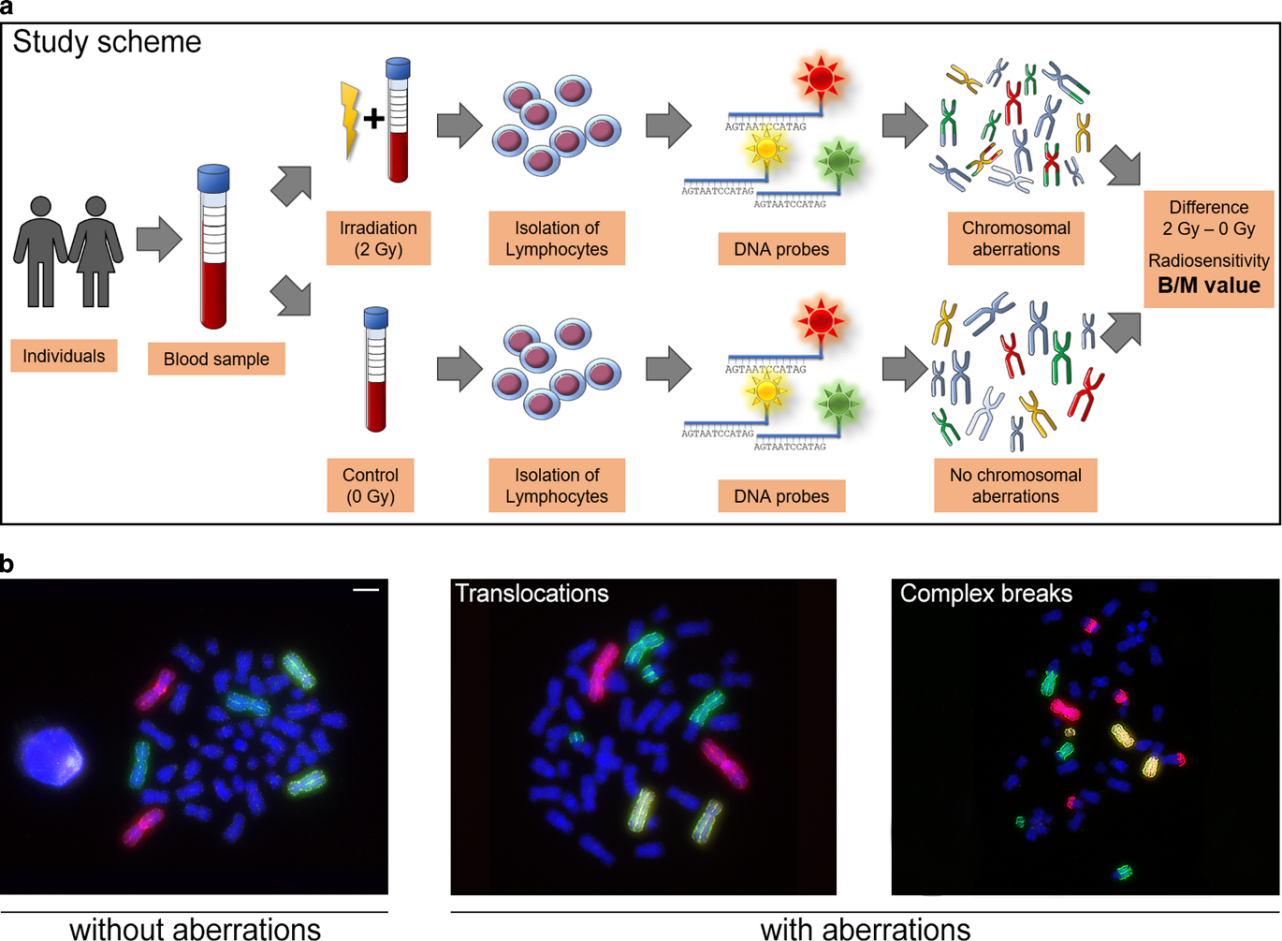
Study scheme and analysis of chromosomal aberrations. **a** Patient blood was collected and processed using a standard protocol. Breaks per metaphases (B/M) were calculated by subtracting the 0 Gy background from the 2 Gy values. **b** Microscopic images after fluorescence in situ hybridization of isolated patient blood lymphocytes. *red* chromosome #1, *green *chromosome #2, *yellow* chromosome #4, *left* chromosomes without aberrations; *middle* translocation of chromosome 1 and chromosome 2, *right* complex chromosomal aberrations. Scale 10 µm

Individual radiosensitivity was studied with peripheral blood lymphocytes and three-color fluorescence in situ hybridization (3C-FISH) to detect chromosomal aberrations as described previously [15, 20]. In brief, individual radiosensitivity was determined in freshly drawn heparinized peripheral blood from cancer patients or healthy individuals. After dividing the blood sample in two aliquots, one was not irradiated and the other irradiated with a dose of 2.0 Gy. Irradiation was done with a linear accelerator used in clinical routine for patient treatment. Normofractionated irradiation (1.8–2.0 Gy) was used to simulate a clinical routine setting [21]. This dose was previously most suitable for distinguishing radiosensitive patients and healthy individuals relying on the statistical power of evaluation of B/M [22, 23]. Ionizing radiation was generated by the linear accelerator Elektra Versa HDTM (Elektra AB, Stockholm, Sweden). After irradiation, lymphocytes were stimulated with RPMI-1640 (Sigma Aldrich, München, Germany) cell culture medium containing 2.5% phytohemagglutinin (PAN biotech, Aidenbach, Germany) and 15% fetal calf serum (FBS; Merck, Darmstadt, Germany) and cultured for 48 h at +37 °C and 5% CO_2_. Afterwards, mitosis was blocked by adding N-Deacetyl-N-methyl-colchicin (0.09 µL/mL; Merck, Darmstadt, Germany). Chromosome preparation was performed using a mix of 75% methanol (Sigma Aldrich, München, Germany) and 25% acetic acid (Sigma Aldrich, München, Germany) and finally DNA was transferred to glass slides. Slides were treated with RNase (Roche, Penzberg, Germany) and pepsin (Sigma Aldrich, München, Germany) and fixated with buffer containing formaldehyde (Merck, Darmstadt, Germany). For the three-color FISH, DNA was denatured using a formamide-containing puffer (Merck, Darmstadt, Germany) at 72 °C. For the hybridization step, a mixture of probes for chromosomes #1, #2 and #4 was incubated for 72 h at 37 °C in the cell culture incubator. Chromosomal aberrations of chromosome #1 (red), chromosome #2 (green) and chromosome #4 (yellow) were detected and analyzed in a semiautomated manner using Biomas software (Version 4.1 07/2018 MSAB, Erlangen, Germany). For valid analysis, we based our calculation on findings of Keller et al. [23] who scored 150 metaphases for “2 Gy” as the minimum to obtain reliable results. For the unirradiated control (0 Gy), referring to more rare spontaneous aberrations, an appropriate number of more than 150 images of metaphases were analyzed. As the B/M value of “0 Gy” generally underly a hyperbola function near the y‑value “0”, our analyses always include as many pictures (a minimum of 150) as necessary to reach a stable and valid B/M value [6, 23]. Radiosensitivity was studied using 3C-FISH of freshly drawn blood samples. The irradiation-induced aberrations were analyzed by staining chromosomes #1, #2 and #4. These chromosomes represent 22% of the whole genome. Based on the initial work of Savage and Simpson [24], aberrations were scored by the number of underlying chromosomal breakages and accumulated to breaks per metaphases [6, 24], which was implemented for this method by Keller et al. [22]. Keller et al. investigated the predictive power of different aberration types such as translocations, complex aberrations and breaks per metaphases. It was proven that the best distinction in radiosensitive of blood lymphocytes between a healthy cohort (*n* = 11) and a hypersensitive cohort (*n* = 5) is delivered by breaks per metaphases (*p* = 0.002, Mann‑Whitney‑U test). As defined in the previously validated scoring, aberrations as deletion, acentric fragments and open breaks were counted as one break event, whereas translocations, dicentric and ring chromosomes were counted as two break events (Fig. 1a). In addition, insertions were counted as three break events and complex aberrations were evaluated according to how many DNA double-strand breaks would theoretically be needed for their formation. Schuster et al. studied blood of 202 healthy individuals and 393 patients and revealed slight differences of radiosensitivity based on chromosomal aberrations with healthy individuals and cancer patients having values of 0.015 and 0.02, respectively [6]. The final value of individual radiosensitivity was calculated as breaks per metaphases (B/M) after subtracting background rates of the 0 Gy sample to normalize for the individual rate of spontaneous aberrations. Regarding previous studies of Keller et al., the underlying thresholds were evaluated as B/M+3×standard deviation (SD) and validation was done empirically over our whole collected data on patient blood. Values of 0.5–0.6 B/M can be assumed as increased radiosensitivity, whereas values greater 0.6 B/M indicate a distinctly increased radiosensitivity [22, 25, 26].

### Patient cohorts

The established negative control cohort consisted of 219 healthy individuals and the positive control cohort of 24 radiosensitive patients. The historical comparative cohort consisted of 452 cancer patients. The main tumor entities were rectal (*n* = 212) and breast cancer (*n* = 146), followed by lung cancer (*n* = 49) and head and neck squamous cell cancer (*n* = 30; Table 1). The study cohort consisted of 49 cancer patients with ongoing kinase inhibitor therapy, as part of the maintenance therapy ensuring that the blood withdrawal was performed when a stable (steady-state) kinase inhibitor plasma concentration was reached. This guaranties a higher comparability of the results, based on the constant therapeutically active concentration of the drug in the organism. This cohort mainly consisted of patients with melanoma (*n* = 20), lung (*n* = 13) and renal cancer (*n* = 12). The used kinase inhibitors were the BRAF inhibitors vemurafenib (*n* = 8) and dabrafenib (*n* = 12), the multikinase inhibitors lenvatinib (*n* = 4) and pazopanib (*n* = 12), the EGFR inhibitors osimertinib (*n* = 7) and the ALK inhibitors alectinib (*n* = 3) and crizotinib (*n* = 3). Gender was equally distributed in the “cancer patient” and the “patients with KI” cohorts (*p* = 0.880). Mean age also did not differ with 62 years in the cancer control cohort and 65 years in the KI cohort (*p* ≥ 0.999). Blood samples were taken pre-RT or > 6 months post-RT to avoid bias by radiation on the background values of chromosomal aberrations, whenever possible.

**Table 1:** Patient characteristics

	Healthy individuals (%)	Radiosensitive patients (%)	Cancer patients (%)	Cancer patients with KI (%)
*n*	209	24	452	49
*Gender (%)*				
Male	90 (43)	10 (42)	210 (49)	25 (51)
Female	119 (57)	14 (58)	220 (51)	24 (49)
*Age (years)*				
Median age	52	55	62	65
SD	18	16	13	14
**Inhibitor (*n*)**				
BRAF inhibitors	–	–	–	–
Dabrafenib	–	–	–	12
Vemurafenib	–	–	–	8
Multi tyrosine kinase inhibitor	–	–	–	–
Pazopanib	–	–	–	12
Lenvatinib	–	–	–	4
EGFR inhibitor	–	–	–	–
Osimertinib	–	–	–	7
ALK inhibitors	–	–	–	–
Alectinib	–	–	–	3
Crizotinib	–	–	–	3
**Cancer type (*n*)**				
Rectal	–	1	212	–
Breast	–	8	146	–
Lung	–	1	49	13
HNSCC	–	–	30	3
Melanoma	–	1	8	20
Prostate	–	1	4	–
Others	–	8	3	1
Renal	–	–	–	12
Unknown	–	4	–	–

*SD *standard deviation

### Statistical analysis

GraphPad prism 8 software (San Diego, CA, USA) was used to perform statistical analysis. One/two-tailed Fisher’s exact test was used to analyze the categorial data (without kinase inhibitor and without radiosensitivity), as well as one-tailed Wilcoxon test and Mann–Whitney U test. *P*-value ≤ 0.05 was determined as significant. Graphs were also generated using GraphPad Prism 8 software.

## Results

### Kinase inhibitor-induced radiosensitivity

Three-color FISH analysis of aberrations in chromosomes #1, #2 and #4 were used to measure the number of B/M after irradiation with a 2 Gy dose (Fig. 1a). An increase in chromosomal aberrations points to increased radiosensitivity (Fig. 1b; [27, 28]). Thresholds of 0.5 and 0.6 B/M indicate slightly or clearly increased radiosensitivity, respectively.

**Fig. 2:**
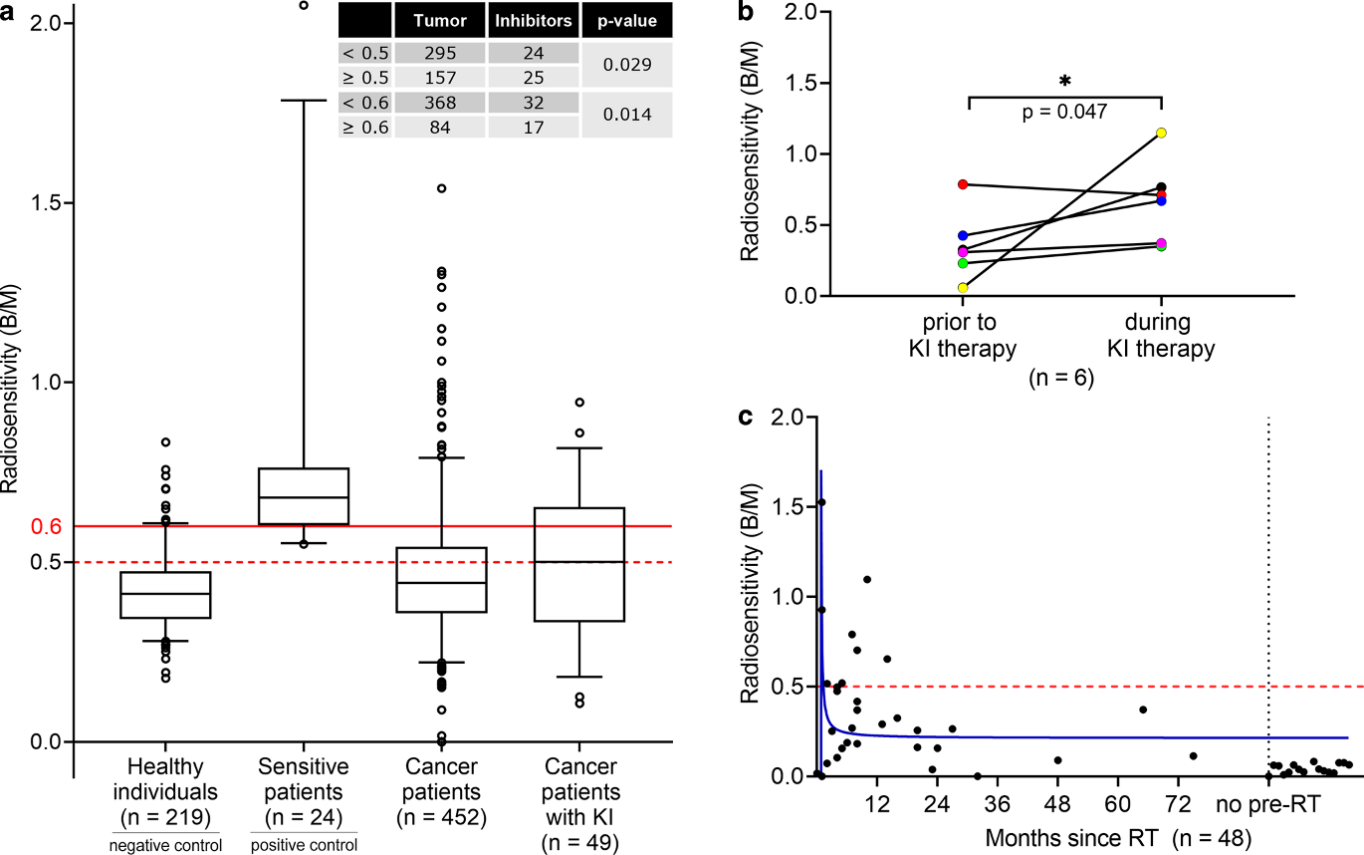
Three-color fluorescence in situ hybridization (FISH) analysis of patient blood under KI treatment. **a** Comparison of a healthy cohort, tumor cohort, sensitive cohort and the KI treatment cohort of all kinase inhibitors. The number of patients with radiosensitivity ≥ 0.5 was significantly increased in KI cohort (two-sided Fisher’s exact test; *p* = 0.0287) as well as values ≥ 0.6 in the KI cohort (two-sided Fisher’s exact test; *p* = 0.0135). **b** Six patients were tested before and during KI treatment. Radiosensitivity (at 2 Gy) was significantly increased (one-tailed Wilcoxon; *p* = 0.0469) during KI therapy. (Treatments: *red, green and purple* pazopanib, *blue *imatinib, *black* alpelisib). **c** Background radiosensitivity (0 Gy) of the tested patients of KI cohort over time after RT (*n* = 48). One patient was excluded because of unclear former therapy

The B/M (mean ± SD) values in lymphocytes of healthy individuals (0.41 ± 0.10) serve as a negative control and a cohort of radiosensitive patients serve as positive control (0.75 ± 0.30). The cancer patient cohort (0.47 ± 0.20) was compared to patients during kinase inhibitor therapy (0.50 ± 0.19; Fig. 2a). Among healthy subjects, 19% had rates ≥ 0.5 B/M and 5% had rates ≥ 0.6 B/M. The highest B/M rates were found in the cohort of sensitive patients, which comprised highly radiosensitive patients who suffered from radiation-related side effects after RT treatment. Increased radiosensitivity was proven, since 100% of all sensitive patients had rates ≥ 0.5 and 79% greater or equal to 0.6 B/M. In the cancer cohort, radiosensitivity was similarly increased as in the group of healthy individuals. Overall, the radiosensitivity of cancer patients was slightly (≥ 0.5) increased in 35% and distinctly (≥ 0.6) increased in 19%. In the cohort with kinase inhibitors, 51% and 35% of all had elevated B/M values ≥ 0.5 and ≥ 0.6, respectively. Compared with the cancer patient cohort, this was a significant increase in the fraction of KI patients with increased B/M values ≥ 0.5 (*p* = 0.029) and ≥ 0.6 (*p* = 0.014).

In a subgroup of 6 patients in the kinase inhibitor cohort, blood samples were available prior to KI therapy and during KI treatment (Fig. 2b). In 5 of these 6 patients individual radiosensitivity increased due to the in vivo kinase inhibitor combined with 2 Gy ex vivo irradiation. It clearly indicates that in vivo kinase inhibitor treatment increases radiosensitivity (*p* = 0.047).

To exclude potential chromosomal instability induced by kinase inhibitors, the unirradiated control samples were analyzed and B/M values were plotted over time after the last RT fraction. There is a clear correlation between the period of time between the end of RT and elevated background levels. B/M values of the unirradiated blood samples (0 Gy) in the kinase inhibitor cohort had background values mostly under the threshold of 0.5 B/M (Fig. 2c). Just 16% of all individuals showed values above 0.5 B/M. There is no evidence that KIs themselves cause chromosomal aberrations. As mentioned in the materials section, this 0 Gy B/M values were subtracted from the 2 Gy values given in Fig. 2a to correct for background.

### Induced radiosensitivity by different kinase inhibitors

**Fig. 3:**
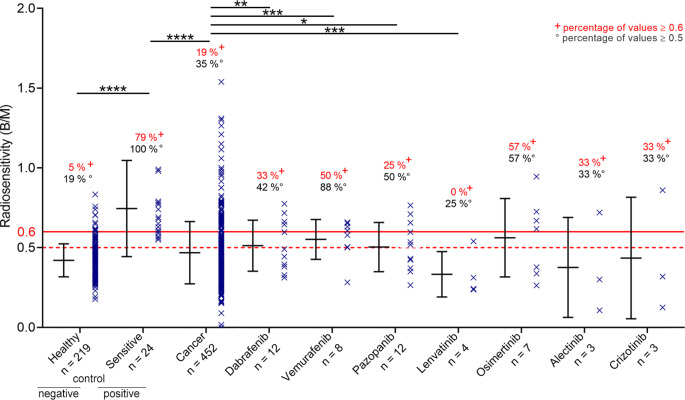
Radiosensitivity of control cohorts and seven different inhibitor cohorts. Radiosensitivity of different cohorts (healthy as positive control, sensitive patients as negative control, tumor patients, and seven different KI-treated groups). The number of patients with radiosensitivity ≥ 0.6 was significantly increased in the sensitive cohort vs. the healthy cohort (two-sided Fisher’s exact test; *p* < 0.0001) and in the sensitive vs. cancer cohort (two-sided Fisher’s exact test; *p* < 0.0001). Subgroups with KI showed increased number of radiosensitive patients for dabrafenib (*p* = 0.0031), vemurafenib (*p* = 0.0005) and pazopanib (*p* = 0.0232). The lenvatinib cohort showed less radiosensitive patients than the cancer cohort (*p* = 0.0005); two-sided Fisher’s exact test. Radiosensitivity was analyzed using three-color FISH and measured in B/M. Cohorts “healthy”, “tumor” and “sensitive patients” were historical control cohorts as published previously [18]. The inhibitor cohorts were collected from patients of the radiation oncology department of the University Hospital Erlangen. Blood samples were collected during KI therapy of the patients

In general, kinase inhibitors significantly increased individual radiosensitivity in ex vivo analyses of blood samples (Fig. 2a). Although we can determine an increase in radiosensitivity in the entire group of the kinase inhibitor cohort, this effect can possibly also be driven by specific drugs. Consequently, distinguishing between the different kinase inhibitors subgroups is necessary (Fig. 3). As mentioned above, the cancer cohort had B/M ≥ 0.6 in 19% of all cases. Radiosensitivity in each subgroup of the seven kinase inhibitors studied yielded B/M values > 0.6 for dabrafenib of 33%, vemurafenib of 50%, pazopanib of 25%, osimertinib 57%, lenvatinib of 0%, alectinib of 33% and crizotinib of 33%. This indicates that increased radiosensitivity can be induced by most of the kinase inhibitors studied.

## Discussion

Kinase inhibitors are able to increase individual radiosensitivity of the tumor and the healthy surrounding tissue and influence the outcome of RT and possible side effects, remarkably. This is known for BRAF inhibitors such as vemurafenib. Increased side effects and cytotoxicity were noticed and therefore pausing the KI treatment is recommended for this group of targeted therapies [8, 18].

Our data suggest that the frequency of radiosensitivity of PMBCs in patients is significantly increased in the KI cohort, which may lead to an increased risk of side effects in normal tissue. Patients during KI therapy showed a gain of B/M compared to the cancer patient cohort in the 3C FISH analysis. Nevertheless, the KI cohort includes different tumor entities and seven different FDA-approved kinase inhibitors. To answer the question whether one KI is reasonable for the rise of B/M values itself, we analyzed the seven KI dabrafenib, vemurafenib, pazopanib, lenvatinib, osimertinib, alectinib and crizotinib separately. BRAF inhibitors dabrafenib and vemurafenib are known to have radiosensitizing potential, which correlates with our data [7, 10, 29, 30]. Occasionally, individual differences in side effects such as radiodermatitis and further severe side effects could be related to interindividual polymorphisms of cancer patients, confirming the range of B/M values [31]. Multikinase inhibitor pazopanib showed less radiosensitizing potential regarding values ≥ 0.6 B/M and lenvatinib, which also targets multiple kinases, showed no distinct radiosensitization ≥ 0.6 B/M. However, several case reports for VEGF inhibitors including pazopanib reported elevated risks such as gastrointestinal perforation and others [32, 33]. Overall, beside occasional cases of hepatotoxicity [34], RT + pazopanib shows good tolerability [35]. No data are available describing interactions between IR and lenvatinib. Noticeable, even in the smallest cohorts of the ALK/ROS and EGFR inhibitors alectinib, crizotinib and osimertinib, which are approved for NSCLC, we were able to find patients with highly increased sensitivity to radiation in PBMCs which should lead to a certain attention. For osimertinib no case reports or clinical data are available. However, increase of sensitivity to radiation is found on the cellular level by delaying DNA damage repair [36]. Effects of alectinib and crizotinib in combination with RT are still discussed controversially, since there are results for both radioprotecting and radiosensitizing ability in NSCLC [37–39]. These diverse outcomes corroborate our thesis that there are interindividual differences in radiosensitivity in cancer patients, which hints at the need to monitor patients particularly closely during RT or if possible, testing every case.

There are a few limitations of our study mainly that blood samples were irradiated and analyzed, exclusively. Basically, the number of patients treated with KI in our clinic is currently low, which is why large cohorts are difficult to collect. Nevertheless, there is noticeable evidence for the correlation of radiosensitivity and the outcome of our FISH analysis of radiosensitivity of PBMCs [26]. In addition, the number of radiosensitive individuals was increased in the cancer cohort than compared to the healthy control group [6]. Different types of medications are able to increase radiosensitivity. Anti-HIV drugs can also increase individual radiosensitivity [15]. Especially the drug efavirenz is known to induce double strand breaks via the induction of cellular oxidative stress [40]. Several kinase inhibitors such as BRAF inhibitors vemurafenib and dabrafenib as well as phosphoinositide 3-kinase inhibitor idelalisib are able to increase radiosensitivity, too [8, 18]. Previous studies were able to show more favorable survival and less toxicity with BRAFi interruption during RT [7]. Furthermore, only small patient cohorts treated with the different KIs were included in our study. Thus, more patients treated with the same inhibitor are needed to validate our data, especially relating blood samples of patients before KI treatment and under sustained maintenance KI therapy post-RT. This type of cohort might be advantageous, since the B/M values show strong interindividual differences. Those strong differences could be mitigated by expanding this group of patients. Also, other commonly used kinase inhibitors such as gefitinib or erlotinib should be included in this patient-related study. It is of certain interest to generate larger groups of the same tumor entity to find reliable correlations between radiosensitivity in healthy tissue and specific kinase inhibitors and irradiation schemes including multiple doses related to clinical fractionation should be assessed for follow-up projects. However, in contrast to previous data, we were able to examine a total of 744 blood samples with the same method. Previous research mainly focused on individual cases (case reports) or small case series of cancer patients [18, 32].

Although kinase inhibitors seem to increase radiation sensitivity, this must not be a disadvantage in the treatment of cancer patients. If kinase inhibitors influence cellular radiation sensitivity in the blood, this effect could lead to increased local tumor control, too. This is an advantage when modern radiotherapeutic methods are used to irradiate more and more precisely and thus less normal tissue is at risk. There is clear evidence that there is a relationship between radiosensitivity of blood and tissue in mice (Rübe et al. 2008), which makes lymphocytes an appropriate representative for healthy tissue. Individual radiosensitivity evaluated by using in vitro irradiated patient-derived blood lymphocytes has been found to correlate with normal tissue reactions [41–44]. Noticeably, an early case report demonstrated complete remission after dramatic dose reduction (1.8 to 0.6 Gy) in an 11-year-old boy diagnosed with ataxia telangiectasia [45]. In addition, blood samples can easily be incorporated into everyday clinical practice and are available in a patient-friendly manner. Sensitizing the tumors of patients for radiation therapy could be an advantage for radiation oncology, when monitoring the patients closely for possible side effects. KI targeting the DNA damage repair proteins PARP1 and PARP2, as well as DNA-PK, ATM and ATR seem to be promising targets for combination with radiation therapy. On the cellular level there is evidence that cytotoxicity can be increased with combination therapy in cancer cells [46–48]. Even though there is an increased risk of skin toxicity with combined therapy using BRAF inhibitors, it is usually well tolerated by most patients [30].

Different approaches are commonly used to analyze radiosensitivity. Cell death, colony forming ability, pathways regarding DNA damage repair and related proteins can be therefore targeted. These assays are easy to establish and use for a clinical approach, but mainly look at only one aspect of cellular radiosensitivity. Using three-color FISH analysis for measuring chromosomal aberrations harbors different advantages compared to more simple assays. The FISH approach uses lymphocytes blocked at the late G2 phase. Therefore, they had to undergo an almost complete cell cycle. Occurrence of cell death, inability to overcome checkpoints and lack of potent DNA repair are covered by this procedure. Taken together, we are able to cover a wide range of possible effects of radiation, leading to a highly reliable indicator of individual radiosensitivity. Nevertheless, the combination of several approaches, e.g., FACS analysis of PBMCs or whole blood samples, should be considered in the future to back our conclusions.

## Conclusions

In 6 out of 7 KIs, we found patients with B/M ≥ 0.6, even within groups of small numbers of cases. In total 17 patients, out of 49, treated with kinase inhibitor showed B/M values ≥ 0.6, which represents 35% of all KI patients. Remarkably, kinase inhibitors do not induce chromosomal aberrations by themselves. Higher B/M values in the 0 Gy samples were related to a short period of time between the blood collection and a previous irradiation. Which rules out that chromosomal instability is not induced by drug treatment, but mainly related to the previous radio(chemo)therapy. Finally, there is evidence for interactions between irradiation and small molecules and correlations of improved overall survival after combination therapy were published in renal cell carcinoma and melanoma brain metastasis [49, 50]. Therefore, radiation therapists should give attention to these findings and common clinical use of radiosensitivity testing should be further developed. Ideally, as a consequence of our data presented in this study, all patients should undergo a close clinical monitoring during concomitant kinase inhibitor and RT. Concerning the up-scaling capability more knowledge about identifying subgroups is needed from distinct clinical trials focusing on simultaneous treatment of irradiation and kinase inhibitors and further more specific related entities harboring more risks or gaining benefit form combination therapy. The development of evidence-based recommendations regarding treatment interruptions or dose adaptions of KI during RT regarding risk of severe side effects on healthy tissue should be a major focus of research to guide individual radiation oncologists in clinical routine.
